# Occipito-transtentorial approach for falcotentorial meningiomas: how I do it

**DOI:** 10.1007/s00701-022-05236-4

**Published:** 2022-05-14

**Authors:** Kyriakos Papadimitriou, Giulia Cossu, Alda Rocca, Roy Thomas Daniel

**Affiliations:** Department of Clinical Neurosciences, Service of Neurosurgery, Lausanne University Hospital and University of Lausanne, CHUV, Rue du Bugnon 46, CH-1011 Lausanne, Switzerland

**Keywords:** Falcotentorial meningiomas, Posterior incisura space

## Abstract

**Background:**

Falcotentorial meningiomas are rare tumors that arise at the junction of the dural folds of the tentorium and falx cerebri, at the junction of the vein of Galen with the straight sinus with possible extensions along the course of the straight sinus. Surgery of falcotentorial meningiomas remains challenging due to the intimate neurovascular relationships in the posterior incisural space.

**Methods:**

We describe the key steps of the occipito-transtentorial approach for falcotentorial meningiomas with a video illustration. The surgical anatomy is described along with the advantages and limitations of this approach.

**Conclusion:**

The occipito-transtentorial approach offers good surgical exposure and outcomes in carefully selected patients harboring falcotentorial meningiomas. Precise understanding of the relationship between the tumor and the internal cerebral veins, basal veins, and vein of Galen should be thoroughly analyzed as these structures may be infiltrated or displaced.

**Supplementary Information:**

The online version contains supplementary material available at 10.1007/s00701-022-05236-4.

## Relevant surgical anatomy

Falcotentorial meningiomas are rare tumors that arise at the junction of the dural folds of the tentorium and falx cerebri, at the junction of the vein of Galen with the straight sinus with possible extensions along the course of the straight sinus [1, 5, 6]. The tumors are located in the posterior incisural space, which lies posterior to the midbrain and extends to the level of the tentorial apex.

As defined by its name, the tentorium and the falx are the sites of primary origin of these rare meningiomas. The tentorium slopes downward from its apex, to its attachment to the temporal, occipital, and sphenoid bones [7]. The lateral and posterior borders enclose the transverse sinus and the torcula. The anterior end of each free edge is attached to the petrous apex and then to the anterior and posterior clinoid processes through the anterior and posterior petroclinoid folds respectively. The falx cerebri is a vertical fold in the midline enclosing the superior sagittal sinus superiorly and then fusing inferiorly into the dorsal surface of the tentorium behind its apex, to enclose the straight sinus at the falcotentorial junction.

The arachnoidal anatomy in the posterior incisural space consists of two membranes that form an arachnoid complex similar to the Liliequist membrane in an inverted fashion [9]. The upper membrane is the posterior perimesencephalic membrane and the lower one is the cerebellar precentral membrane which is similar to the diencephalic component of the membrane of Liliequist. A thinner component, the intracrural membrane extends from the posterior part of the uncus to the cerebral peduncle and adjacent part of the optic tract [3].

The posterior incisural space has an anterior wall, a roof, and lateral walls. The anterior wall consists from superiorly to inferiorly by the pineal body rostrally, the quadrigeminal plate and by the lingula of the vermis on the midline [7]. The roof is formed by the lower surface of the splenium, the crura of the fornices, and the hippocampal commissure. Each lateral wall is formed by the pulvinar, the crus of the fornix, the medial surface of the posterior part of the parahippocampal and dentate gyri [7].

The quadrigeminal cistern is included in the posterior incisural space, situated just posterior to the quadrigeminal plate. It communicates superiorly with the posterior pericallosal cistern, inferiorly with the cerebellomesencephalic fissure, inferolaterally with the posterior part of the ambient cistern [7]. The posterior portion of the third ventricle is just anterior to the posterior incisural space, while the atria and occipital horns of the lateral ventricles are situated at a larger distance laterally.

The arterial anatomy of the posterior incisural space consists of the posterior cerebral artery (PCA) and superior cerebellar artery (SCA) as they enter this space in an antero-posterior direction. The PCA is situated in the lateral part of the posterior incisural space and gives rise to the lateral and medial posterior choroidal arteries before bifurcating into the calcarine and parieto-occipital arteries. The SCA branches pass below the free edge of the tentorium to supply the tentorial surface of the cerebellum [7].

The venous anatomy of the posterior incisura space is composed of the two internal cerebral veins and the basal veins that exit the velum interpositum and the ambient cistern, respectively, to reach the posterior incisural space where they join to form the vein of Galen [5–7]. The site of the junction of the vein of Galen with the straight sinus can vary. It can be flat if the tentorial apex is located below the splenium or it can form a sharp angle if the apex is located above the splenium, so that the vein of Galen turns sharply upward to reach the straight sinus [6].

## Description of the technique

The patient is positioned in the park bench position with the head immobilized on a Mayfield headclamp with the head flexed, tilted upward and turned to the contralateral side. This would place the craniotomy site positioned toward the floor allowing the ipsilateral occipital lobe to be retracted aided by gravity and the contralateral occipital lobe kept in place away from the craniotomy. A lumbar drain is positioned before starting the procedure to obtain brain relaxation during the interhemispheric approach. An inverted “U” incision, centered on the occipital and suboccipital region is performed, with a vertical branch of the “U” on the midline. Using standard anatomical landmarks, the trajectory of the posterior part of the superior sagittal sinus (SSS), the torcular, and the transverse is identified. The occipital artery should be spared during muscle dissection to preserve the vascularization of the musculo-cutaneous flap.

**Fig. 1:**
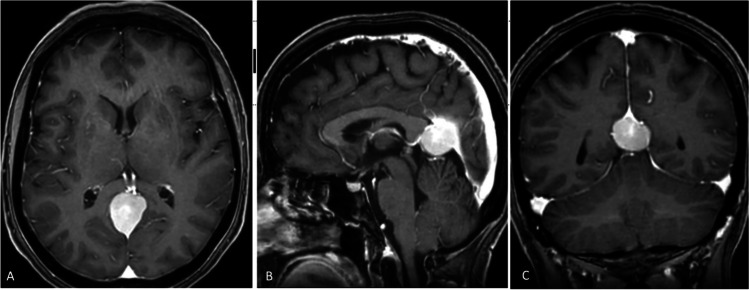
A 57-year-old female without any past medical history presented to our institution with headache and neck pain for the last 6 months. She did not have any other neurological signs or symptoms. A full neurological examination was within normal limits. Cranio-cervical MRI was performed, which revealed a 30 × 25 × 20 mm extra-axial tumor compatible with meningioma. **A** axial, **B** sagittal, and **C** coronal T1 contrast enhanced MRI. The tumor was located at the anterior part of falcotentorial junction projecting into the pineal region and found displacing the deep venous system upwards. Notably, the inferior sagittal sinus, the vein of Galen and the straight sinus were patent. At surgery we confirmed the fact that the primary origin of the tumor was from the tentorium extending to the falcotentorial junction, posterior part of inferior sagittal sinus and anterior part of the straight sinus (type T3 Yasargil’s classification) [8]

The craniotomy should expose the lateral part of SSS, the torcula, and the superior portion of the transverse sinus. The sinuses are skeletonized and protected with wet gelfoam. A 5–8 mm durotomy parallel to the SSS and just lateral to it is performed, to avoid exposition of the brain. Dural hitch stitches allow gentle retraction of the sinus to improve vision. Evacuation of approximately 15–20 cc of CSF through the lumbar drain offers brain relaxation. An interhemispheric occipital approach should be performed in accordance with the venous anatomy, to respect the cortical bridging veins going to the SSS. This allows exposition of the falx (medially) and the ipsilateral tentorium inferiorly. Using navigation, the trajectory of the straight sinus is defined which is often displaced upwards by the tumor (Fig. 1).

**Fig. 2:**
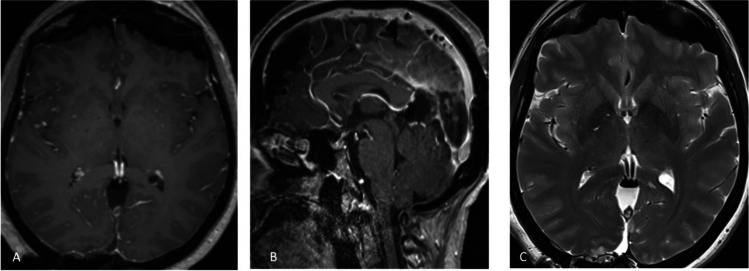
Post-operative T1-contrast enhanced MRI (**A**) axial and (**B**) sagittal images, showed complete resection with patent deep venous system and straight sinus. T2-weighted MRI did not reveal any complications such as occipital lobe edema (**C**)

The trajectory of the straight sinus is confirmed utilizing intra-operative navigation. Alternatively, indocyanine green videography can be used to identify the straight sinus.The ipsilateral tentorium is cut lateral to the tumor upto the tentorial hiatus. The falx is cut superior to the straight sinus to allow visualization of the contralateral dorsal surface of the tumor. Through this falcine incision, the tentorium is incised on the contralateral side lateral to the tumor margin and extended to the tentorial hiatus. This allows devascularisation of the tumor, following which, progressive debulking of the tumor is achieved with ultrasonic aspiration. The debulking is carried on until the arachnoid planes on the surface of the tumor are identified. The attachment of the tumor to the inferior sagittal and straight sinuses is disconnected after ensuring patency of the deep venous system. Preservation of the arachnoidal planes allows the integrity of the pre-central cerebellar vein inferiorly and the deep veins superiorly. At the end of the resection, the internal cerebral veins, as well as the vein of Galen, are visualized through a thin arachnoid layer (Fig. 2). The dura is closed watertight and the bone flap is positioned. The surgical wound is closed in layers.

## Indications for surgery

The occipital transtentorial approach was initially described by Poppen to access lesions to the pineal region, and other deep-seated tumors in the quadrigeminal cistern [5]. Surgical resection is indicated for patients who have enlarging tumors causing progressive neurologic deficit, such as visual deficits, fourth cranial nerve deficit, and/or signs and symptoms of intracranial hypertension usually as a result of hydrocephalus [10]. On the other hand, calcified tumors in older patients that are stable should be observed with serial imaging.

## Limitations of the occipito-transtentorial approach (OTA)

This approach can be associated with prolonged occipital lobe retraction which may be a causative factor for visual deficits. The Herophilus-Galen line represents the limit of sight of the microscope. In contrast to the supracerebellar-infratentorial approach (SIA), which is considered the alternative approach, in order to gain access to the contralateral half of the quandrigeminal cistern, a transfalcine approach is needed [2]. Moreover, the galenic system is encountered earlier compared to the SIA. Injury to any tentorial sinuses should be avoided during the tentorial incision. The combined bilateral occipital transtentorial/transfalcine technique has been proposed for larger tumors which have most often occluded the straight sinus [6].

## How to avoid complications

Using gravity-assisted occipital lobe retraction, combined with limited use of retractors and intermittent CSF drainage through a lumbar drain, helps avoid retraction injury [4]. Sinuses skeletonization should be carefully performed with the use of diamond drills in order to avoid sinus injury. They should be covered by wet Gelfoam to limit the risk of thrombosis and protection from thermal injury. The arachnoid layer covering the deep venous structures should not be violated to avoid postoperative thrombosis or inadvertent injuries. Venous injury or postoperative deep vein thrombosis can have catastrophic neurological consequences.

## Preoperative workup

Precise preoperative planning with special consideration to the location and permeability of deep venous drainage system is imperative. The relationship between the tumor and the internal cerebral veins, basal veins, and vein of Galen should be thoroughly analyzed as these structures may be infiltrated or displaced. Preoperative brain MRI and computed tomography (CT) scan with bone windows are performed to study tumor anatomy, potential calcifications and its relationships to adjacent structures. MR venography allows a clear delineation of the relationships of the tumor with respect to the deep venous system in addition to the superficial venous system that needs to be studied for the craniotomy and approach. Moreover, MR angiography allows assessment of the degree of occlusion of the straight sinus and, in giant tumors, of the torcula and transverse sinus. Neuro-opthalmological examinations are indicated in large tumors and for those cases where a occipito-transtentorial approach is considered.

## Postoperative workup

Postoperative MRI is performed to verify the extent of resection and permeability of all the deep and superficial venous systems. MRI is performed at 3 months follow-up to look for any residual tumor attachments. Neuro-opthalmological examinations are performed to detect any visual deficits related to occipital lobe mobilization.

## Instructions for the postoperative care

The patient is monitored in the intermediate care unit for at least 24 h with regular hemodynamic and neurological check-ups. Neurological examination is performed in order to detect any potential complications related to surgery. The patient is kept well hydrated to avoid any deep venous or sinus thrombosis.

## Specific information to give to the patient about surgery and potential risks

The most frequent complications are hemorrhage, wound infections, hydrocephalus, occipital lobe ischemia or retraction injury which can lead to visual field deficits. Recurrent meningiomas or growing residual tumors may necessitate adjuvant treatment, such as radiosurgery [9].

## Supplementary Information

Below is the link to the electronic supplementary material.

Supplementary file1 (MP4 161303 KB)
